# Indomethacin Disrupts the Formation of β-Amyloid Plaques via an α2-Macroglobulin-Activating lrp1-Dependent Mechanism

**DOI:** 10.3390/ijms22158185

**Published:** 2021-07-30

**Authors:** Pei-Pei Guan, Liu-Qing Yang, Guo-Biao Xu, Pu Wang

**Affiliations:** College of Life and Health Sciences, Northeastern University, Shenyang 110819, China; guanpp@mail.neu.edu.cn (P.-P.G.); lqyang0202@163.com (L.-Q.Y.); xugb_bio@163.com (G.-B.X.)

**Keywords:** Alzheimer’s disease, indomethacin, α_2_-macroglobulin, low-density lipoprotein receptor-related protein 1, prostaglandin D_2_ receptor 2, brain efflux of β-amyloid protein

## Abstract

Epidemiological studies have implied that the nonsteroidal anti-inflammatory drug (NSAID) indomethacin slows the development and progression of Alzheimer’s disease (AD). However, the underlying mechanisms are notably understudied. Using a chimeric mouse/human amyloid precursor protein (Mo/HuAPP695swe) and a mutant human presenilin 1 (PS1-dE9) (APP/PS1) expressing transgenic (Tg) mice and neuroblastoma (N) 2a cells as in vivo and in vitro models, we revealed the mechanisms of indomethacin in ameliorating the cognitive decline of AD. By screening AD-associated genes, we observed that a marked increase in the expression of α_2_-macroglobulin (A2M) was markedly induced after treatment with indomethacin. Mechanistically, upregulation of A2M was caused by the inhibition of cyclooxygenase-2 (COX-2) and lipocalin-type prostaglandin D synthase (L-PGDS), which are responsible for the synthesis of prostaglandin (PG)H_2_ and PGD_2_, respectively. The reduction in PGD_2_ levels induced by indomethacin alleviated the suppression of A2M expression through a PGD_2_ receptor 2 (CRTH2)-dependent mechanism. Highly activated A2M not only disrupted the production and aggregation of β-amyloid protein (Aβ) but also induced Aβ efflux from the brain. More interestingly, indomethacin decreased the degradation of the A2M receptor, low-density lipoprotein receptor-related protein 1 (LRP1), which facilitated the brain efflux of Aβ. Through the aforementioned mechanisms, indomethacin ameliorated cognitive decline in APP/PS1 Tg mice by decreasing Aβ production and clearing Aβ from the brains of AD mice.

## 1. Introduction

Alzheimer’s disease (AD) is the most diagnosed neurodegenerative disease that is considered to increase in dementia with age [1]. Most current investigations ascribe the underlying causes of AD to the deposition of β-amyloid protein (Aβ) and hyperphosphorylation of tau, resulting in the formation of β-amyloid plaques (APs) and neurofibrillary tangles (NFTs) [2]. The formation of APs and NFTs progressively induces synaptic dysfunction, apoptosis and neuronal death in the brains of patients with AD, especially in the hippocampus [3,4]. Extensive research has described an association between neuroinflammation and AD [5]. Indeed, neuroinflammation has been regarded as a prominent feature of AD brain tissues that accompanies the development and progression of the disease [6].

A series of studies proposed protective effects of nonsteroidal anti-inflammatory drugs (NSAIDs) on AD [7]. For example, aspirin obviously inhibits the formation of β-sheet structures of Aβ_25–35_, Aβ_1–40_ and Aβ_1–42_. In addition to aspirin, indomethacin, naproxen, ketoprofen, ibuprofen, celecoxib and rofecoxib also disrupt the aggregation of Aβ [8]. Apart from their inhibitory effects on the aggregation of Aβ, NSAIDs, including indomethacin, ibuprofen, sulindac and flurbiprofen, have been reported to reduce the levels of Aβ in cultured cells [9,10,11]. Consistent with these in vitro observations, NSAIDs, including ibuprofen and nitric oxide (NO)-releasing derivatives of flurbiprofen, such as HCT1026 and NCX2216, decrease the production and deposition of Aβ in chimeric mouse/human amyloid precursor protein (Mo/HuAPP695swe) and a mutant human presenilin 1 (PS1-dE9) (APP/PS1) and Tg2576 mice [12]. In particular, NCX 2216 reduces the overloading of Aβ in the cerebral cortex to a greater extent than ibuprofen when administered to Tg2576-crossed PS1^M146L^ transgenic (Tg) mice for 5 months [12]. Other NSAIDs, including indomethacin and *R*-flurbiprofen, inhibit the pathology of Aβ in Tg2576 mice [13,14].

As a classical NSAID, indomethacin exerts positive effects on clearing Aβ and improving the cognitive decline of AD experimental models. In Tg2576 mice, 2.24 mg/kg/d indomethacin treatment for 8 months significantly decreased the overloading of Aβ [13]. In addition, 10 mg/kg/d indomethacin treatment reduced the levels of both Aβ_1–42_ and Aβ_1-40_ in Tg2576 mice [15]. In addition to its inhibitory effects on the production of Aβ, indomethacin exerts protective effects on memory in rats with electroconvulsive shock-induced retrograde amnesia [16]. More importantly, treatment with indomethacin and atorvastatin ameliorates the cognitive decline of APP/PS1 Tg mice [17]. Due to its anti-inflammatory effects, indomethacin reverses the activity of the microglia surrounding Aβ deposits after an infusion into the lateral ventricles of rats [18]. Specifically, multiple proinflammatory factors, including nuclear factor (NF)-κB and inducible nitric oxide synthase (iNOS), are also inhibited by treatment with indomethacin both in vivo and in vitro [19,20].

Similar to the animal studies, 100 to 150 mg/d indomethacin seemed to be beneficial for patients with AD and mild to moderate cognitive decline in a clinical trial [21]. According to a battery of cognitive tests, indomethacin-treated patients improved by 1.3 ± 1.8%, whereas placebo-treated patients exhibited an 8.4 ± 2.3% decrease, resulting in a significant difference (*p* < 0.003) [21]. Due to the well-known gastrointestinal side effects of indomethacin, a substantial proportion of patients with mild AD treated with this drug dropped out of the clinical trial, resulting in no significant difference between the indomethacin and control groups, especially in scores on the Mini-Mental State Examination, AD assessment scales, Boston naming tests and token tests [21]. In a one-year randomized controlled clinical trial, indomethacin did affect AD progression [22]. The failure of these clinical trials might be caused by the short-term administration of indomethacin. In support of this hypothesis, the adjusted odds ratios for AD among indomethacin users decreased from 0.98 for ≤1 year of use (95% CI 0.91–1.03) to 0.45 for >5 years of use (0.13–1.55) in a clinical trial of 49,349 patients with AD and 196,850 matched controls [23]. In addition, NSAIDs prevented but did not reverse neuronal cell cycle reentry in a mouse model of AD [24]. This finding might be another explanation for the failure of clinical trials, since most are performed with patients at the late stage of AD. Subsequently, DP-155, a lecithin derivative of indomethacin, was designed and synthesized to reduce gastrointestinal and renal toxicity [25]. After repeated oral dosing, gastrointestinal and renal toxicity was lower in individuals treated with DP-155 compared to those treated with indomethacin [25]. If side effects were not considered, DP-155 and indomethacin exerted similar effects on decreasing Aβ overload in the brains of Tg2576 mice [25]. Unfortunately, the development of this indomethacin derivative has been interrupted.

As a nonspecific inhibitor of cyclooxygenase (COX) [26], indomethacin inhibits the activity of the enzyme prostaglandin (PG) H synthase, which is responsible for the synthesis of PGH_2_, a precursor of various PGs and thromboxane [27]. COX exists in two isoforms, COX-1 and COX-2, with high structural identity. However, their substrates, inhibitor selectivity and intracellular localization are distinct [27]. COX-1 is constitutively expressed in many cell types and is presumed to be responsible for the synthesis of housekeeping PGs. In contrast, COX-2 is either absent or minimally expressed in most normal tissues and increases during inflammatory responses [27]. Thus, researchers have assumed that COX-2 expression is primarily increased at sites of disease and inflammation. Based on these previous studies, much attention has focused on the role of COX-2 in AD pathogenesis. For instance, overexpression of COX-2 was reported to aggravate the pathogenesis of AD [28]. In addition, metabolic products, such as prostaglandin (PG) E_2_, PGD_2_ and PGI_2_, are involved in regulating the development and progression of AD by mediating the deposition of Aβ and hyperphosphorylated tau [29,30,31]. However, researchers have not yet clearly determined whether COX-2 is involved in regulating the transport of Aβ in the brains of patients with AD.

Interestingly, the human proteinase inhibitor α2-macroglobulin (A2M) has been suggested as being involved in the synthesis of COX-2 [32] and its metabolic product, PGE_2_, in macrophages [33]. In addition, exposure of murine macrophages to A2M results in the secretion of eicosanoids, which potentially contributes to PG synthesis in a COX-2-dependent manner [34]. Reciprocally, a relationship between PGE_2_ and A2M in gingival crevicular fluid was also established in adults with chronic periodontitis [35]. Although their relationship has not been explored in AD, A2M has been regarded as being involved in the pathogenesis of the disease. A2M binds to Aβ to form heterodimers [36]. The formation of A2M and Aβ complexes prevents the formation of Aβ fibrils [37]. More importantly, stable A2M-Aβ complexes are recognized and cleared by the A2M receptor/low-density lipoprotein-related receptor [A2M R/low-density lipoprotein receptor-related protein 1 (LRP)] in the brain [36].

Although A2M and LRP are regarded as proteins that effectively clear Aβ, neither their upstream regulators nor stimulators have been identified to elucidate the relevant mechanisms involved in these interactions. Therefore, the current study aims to establish the relationship between indomethacin and A2M, through which the cognitive decline of AD is improved via modulating the production and aggregation of Aβ and inducing Aβ efflux in an LRP1-dependent manner.

## 2. Results

### 2.1. Indomethacin Improves Cognitive Decline in APP/PS1 Tg Mice

**Figure 1:**
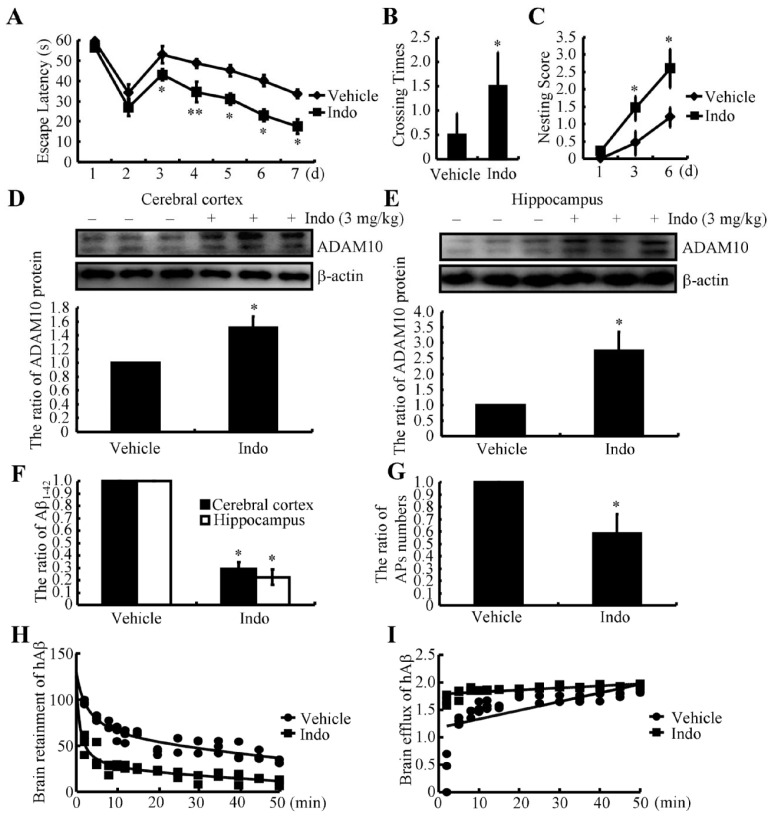
Indomethacin attenuates cognitive decline in APP/PS1 Tg mice by increasing ADAM10 expression, resulting in decreased production and deposition of Aβ and inducing Aβ efflux in vivo. (**A**–**I**) Six-month-old APP/PS1 mice were orally administered indomethacin (3 mg/kg/d) for 3 months before their learning ability was assessed using the Morris water maze test or the nest construction assay. (**A**) Escape latency of mice in the visible and hidden platform experiments. (**B**) In the spatial exploration experiment, the number of times the animals crossed over the original platform was recorded by the software. (**C**) Nest scores of different groups of mice analyzed using the nest construction assay. After determining learning ability, the brains of mice were collected and separated into the cerebral cortex and hippocampus. (**D**,**E**) Western blotting was employed to detect the expression of ADAM10. Β-actin served as an internal control. The optical density of the bands was analyzed using ImageJ software (v1.51, NIH, Bethesda, MD, USA). (**F**) The content of Aβ_1–42_ in the cerebral cortex and hippocampus of APP/PS1 Tg mice was detected using an ELISA. (**G**) The distribution of APs in the cerebral cortex and hippocampus of APP/PS1 Tg mice was determined using IHC and analyzed microscopically. (**H**,**I**) *hAβ* (2 μg/5 μL) was injected (i.c.v.) into the brains of WT mice that were orally pre-administered indomethacin (3 mg/kg) for 2 h. Then, brains were collected at the indicated time points after perfusion. The remaining *hAβ* was analyzed using ELISA. Brain retention and efflux of *hAβ* were calculated as described in the “Materials and Methods”. Data are presented as the means ± S.E. of independent experiments. * *p* < 0.05 and ** *p* < 0.01 compared to vehicle-treated APP/PS1 Tg mice.

Given the potential roles of indomethacin in AD [21], we initially determined its effects on learning ability in APP/PS1 Tg mice. Using the Morris water maze, we observed that indomethacin treatment exerted a limited effect on restoring the learning ability of APP/PS1 Tg mice (Figure 1A–C). During the pretraining visible platform experiments, indomethacin treatment did not produce differences compared to vehicle-treated APP/PS1 Tg mice (Figure 1A), suggesting that neither indomethacin nor the vehicle influenced the mortality or vision of APP/PS1 Tg mice. In subsequent invisible platform tests, indomethacin treatment gradually shortened the time for APP/PS1 Tg mice to find the platform (Figure 1A). On the last day, we removed the platform for a probe test. Untreated mice showed no preference toward the target quadrant, indicating a severe memory impairment, whereas indomethacin-treated APP/PS1 Tg mice exhibited improved behavior compared to untreated controls (Figure 1B). Moreover, nest construction assays were performed to determine the effects of indomethacin on the memory of an inborn ability. Indomethacin treatment increased nesting scores of APP/PS1 Tg mice compared to untreated controls (Figure 1C). Based on these observations, we can conclude that long-term administration of indomethacin is beneficial for improving cognitive decline in APP/PS1 Tg mice.

### 2.2. Indomethacin Suppresses Aβ Production and Deposition by Inducing the Expression of α-Secretases in APP/PS1 Tg Mice

We initially examined the effects of indomethacin on the expression of α-secretase in the cerebral cortex and hippocampus of APP/PS1 Tg mice to reveal the inherent mechanisms underlying its effects. Western blotting revealed that indomethacin treatment clearly increased the expression of ADAM10 in the cerebral cortex and hippocampus of APP/PS1 Tg mice (Figure 1D,E). Regarding the ability of α-secretase to decrease Aβ production, we further examined Aβ_1–42_ levels in the cerebral cortex and hippocampus of APP/PS1 Tg mice. Indomethacin treatment inhibited Aβ_1–42_ production in the brains of APP/PS1 Tg mice (Figure 1F). Moreover, deposition of Aβ in APs was also suppressed in the brains of APP/PS1 Tg mice following indomethacin treatment (Figure 1G). Therefore, indomethacin is beneficial for improving cognitive decline by inhibiting the production and deposition of Aβ in an α-secretase-dependent manner.

### 2.3. Indomethacin Induces Aβ Efflux Both In Vivo and In Vitro

Since indomethacin exerts inhibitory effects on the levels of Aβ, we examined whether indomethacin altered the transportation of Aβ into or out of the brain. For this purpose, we determined the brain retention and efflux of injected (intracerebroventricular injection, i.c.v.) *hAβ* in the absence or presence of indomethacin (per oral, p.o.). Pretreatment with indomethacin (3 mg/kg) for 2 h obviously decreased the brain retention of hAβ in WT mice (Figure 1H). Reciprocally, indomethacin enhanced the efflux of *hAβ* in WT mice (Figure 1I). Based on these observations, we can conclude that indomethacin accelerates Aβ metabolism in mouse brains.

### 2.4. Indomethacin Treatment Increases the Expression of A2M

**Figure 2:**
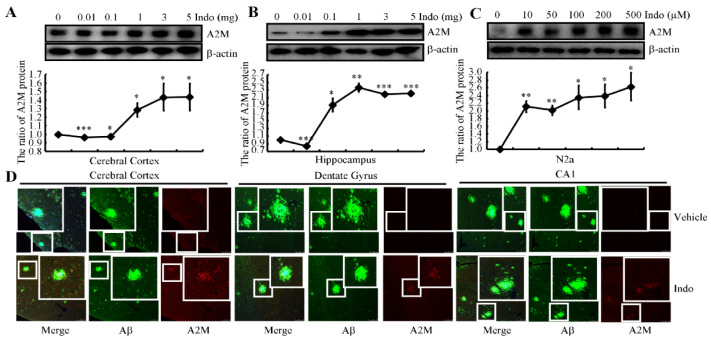
Indomethacin induces the expression of A2M and its colocalization with APs. (**A**,**B**) APP/PS1 Tg mice were intragastrically administered the indicated concentrations of indomethacin ranging from 0.01 to 5 mg/kg. Western blotting was employed to detect the expression of A2M. β-actin served as an internal control. (**C**) N2a cells were treated with the indicated concentrations of indomethacin ranging from 10 to 500 μM for 24 h. Western blotting was used to determine the expression of A2M. β-actin served as an internal control. The optical density of the bands was analyzed using ImageJ software (v1.51, NIH, Bethesda, MD, USA). (**D**) Six-month-old APP/PS1 mice were intragastrically administered indomethacin (3 mg/kg/d) for 3 months before collecting the brains. Brain sections of mice were double-stained with A2M and Aβ antibodies, which were then observed under a confocal microscope. Data are presented as the means ± S.E. of independent experiments. * *p* < 0.05; ** *p* < 0.01 and *** *p* < 0.001 compared to vehicle-treated controls.

Given the ability of indomethacin to induce Aβ efflux, we continued to explore the inherent mechanisms underlying this process. For this purpose, APP/PS1 Tg mice were intragastrically administered the indicated concentrations of indomethacin ranging from 0.01 to 5 mg/kg. As a result, the expression of the A2M protein was markedly increased in the cerebral cortex and hippocampus of APP/PS1 Tg mice administered concentrations of indomethacin greater than 1 mg/kg (Figure 2A,B). We selected 3 mg/kg for subsequent in vivo experiments to ensure the efficacy of indomethacin. We treated N2a cells with the indicated concentrations of indomethacin (from 10 to 500 μM) for 24 h to validate this in vivo result. As shown in Figure 2C, treatment with 10 μM indomethacin markedly increased the expression of the A2M protein in N2a cells, which plateaued at a concentration of 100 μM. To achieve higher induction of A2M, we selected 100 μM indomethacin for subsequent experiments in N2a cells.

A2M binds to Aβ to form heterodimers, resulting in recognition and clearance by the A2M receptor/low-density lipoprotein-related receptor (A2M R/LRP) [36] and preventing the formation of Aβ fibrils in the brain [37]. We therefore double-stained brain slices from 9-month-old APP/PS1 Tg mice with Aβ and A2M antibodies. A2M (red) was enriched in neuronal cells but not APs (green) in the brains of 9-month-old APP/PS1 Tg mice (Figure 2D, upper panel). More interestingly, A2M appeared in APs in indomethacin-treated mice (Figure 2D, lower panel). Based on these observations, it can be concluded that indomethacin not only induces the expression of A2M but also triggers the colocalization of APs and A2M in the brains of APP/PS1 Tg mice.

### 2.5. Indomethacin Inhibits the Expression of COX-2, L-PGDS and CRTH-2

**Figure 3:**
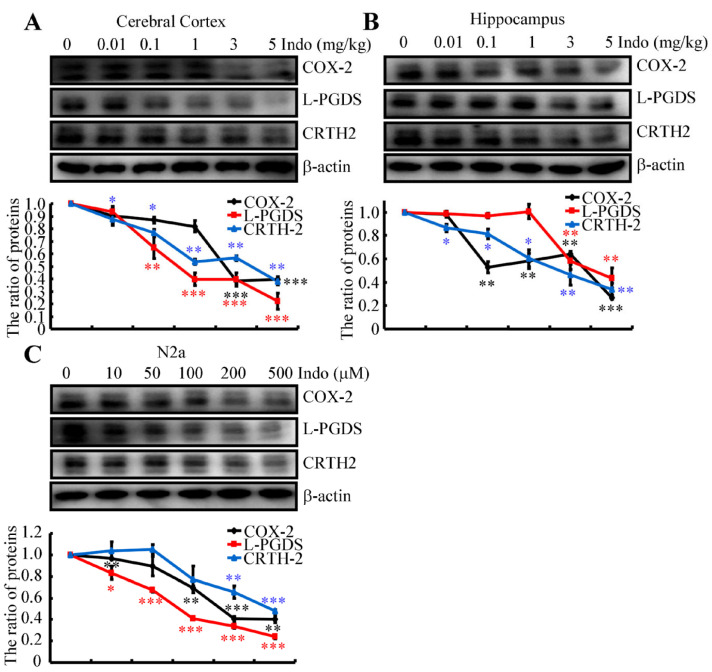
Indomethacin treatment suppresses the expression of COX-2, L-PGDS and CRTH2 proteins. (**A**,**B**) APP/PS1 Tg mice were intragastrically administered the indicated concentrations of indomethacin ranging from 0.01 to 5 mg/kg. Western blotting was employed to detect the levels of COX-2, L-PGDS and CRTH2. β-actin served as an internal control. (**C**) N2a cells were treated with the indicated concentrations of indomethacin ranging from 10 to 500 μM for 24 h. Western blotting was used to determine the expression of COX-2, L-PGDS and CRTH2. β-actin served as an internal control. The optical density of the bands was analyzed using ImageJ software (v1.51, NIH, Bethesda, MD, USA). Data are presented as means ± S.E. of independent experiments. * *p* < 0.05; ** *p* < 0.01 and *** *p* < 0.001 compared to vehicle-treated controls.

As a nonspecific inhibitor of cyclooxygenase (COX) [26], indomethacin inhibits the activity of the enzyme prostaglandin (PG) H synthase, which is responsible for the synthesis of PGH_2_, a precursor of various PGs and thromboxane [27]. As an inducible enzyme, COX-2 protein expression was initially determined in indomethacin-treated APP/PS1 Tg mice using Western blotting. The results show that 3 or 5 mg/kg indomethacin clearly downregulated the expression of the COX-2 protein in the cerebral cortex and hippocampus of APP/PS1 Tg mice (Figure 3A,B). However, the effects of indomethacin on the metabolism of PGH_2_ were not only restricted to COX-2. Interestingly, indomethacin treatment suppressed the expression of PGD_2_ synthase and L-PGDS in the cerebral cortex and hippocampus of APP/PS1 Tg mice (Figure 3A,B). Moreover, the expression of the PGD_2_ receptor CRTH2 was inhibited in the brains of APP/PS1 Tg mice following treatment with indomethacin (Figure 3A,B). N2a cells were treated with the indicated concentrations of indomethacin for 24 h to validate these in vivo observations. Consistent with the in vivo results, the expression of the COX-2, L-PGDS and CRTH2 proteins was downregulated in indomethacin-treated N2a cells (Figure 3C). As the metabolic product of L-PGDS, PGD_2_ synthesis was also suppressed by the indomethacin treatment (Figure 3C). Together, these findings suggest the potential involvement of PGD_2_ signaling cascades in regulating the expression of A2M.

### 2.6. PGD_2_ Suppresses the Expression of the A2M Protein in a CRTH2-Dependent Manner

**Figure 4:**
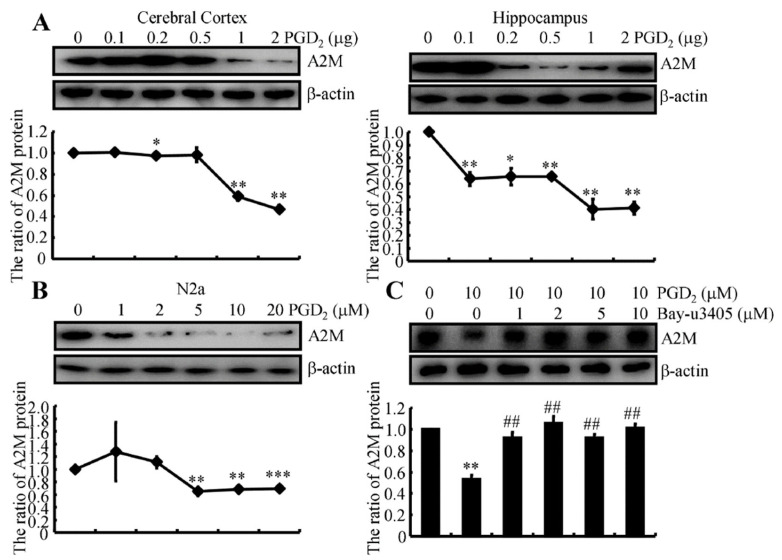
PGD_2_ suppresses the expression of the A2M protein in a CRTH2-dependent manner. (**A**) APP/PS1 Tg mice were injected (i.c.v.) with the indicated doses of PGD_2_ ranging from 0.1 to 2 μg. After 24 h, Western blotting was used to determine the expression of the A2M protein. β-actin served as an internal control. (**B**) N2a cells were treated with the indicated concentration of PGD_2_ from 1 to 20 μM for 24 h. (**C**) In selected experiments, N2a cells were treated with 10 μM PGD_2_ in the absence or presence of the indicated concentrations of a CRTH-2 inhibitor, Bay-u3405, ranging from 1 to 10 μM for 24 h. Western blotting was used to determine the expression of A2M. β-actin served as an internal control. The optical density of the bands was analyzed using ImageJ software (v1.51, NIH, Bethesda, MD, USA). Data are presented as the means ± S.E. of independent experiments. * *p* < 0.05; ** *p* < 0.01 and *** *p* < 0.001 compared to vehicle-treated controls. ## *p* < 0.01 compared to PGD_2_-treated groups.

Based on the hypothesis described above, PGD_2_ was injected (i.c.v.) into APP/PS1 Tg mice to determine the effects of PGD_2_ on the expression of the A2M protein in the cerebral cortex and hippocampus. A relatively high-dose PGD_2_ (>1 μg) injection (i.c.v.) suppressed the expression of A2M protein in the brains of APP/PS1 Tg mice (Figure 4A). These in vivo observations were further reinforced in indomethacin-treated N2a cells (Figure 4B). Mechanistically, PGD_2_ usually exerts its effects through its receptors. By blocking the activity of CRTH2, Bay-u3405 attenuated the effects of PGD_2_ on suppressing the expression of A2M in N2a cells (Figure 4C). Therefore, PGD_2_ inhibits the expression of the A2M protein via a CRTH2-dependent mechanism.

### 2.7. A2M Is Responsible for Inducing ADAM10 Expression via RAP-Dependent Activation of the PI3-K/AKT and ERK1/2 Pathways

**Figure 5:**
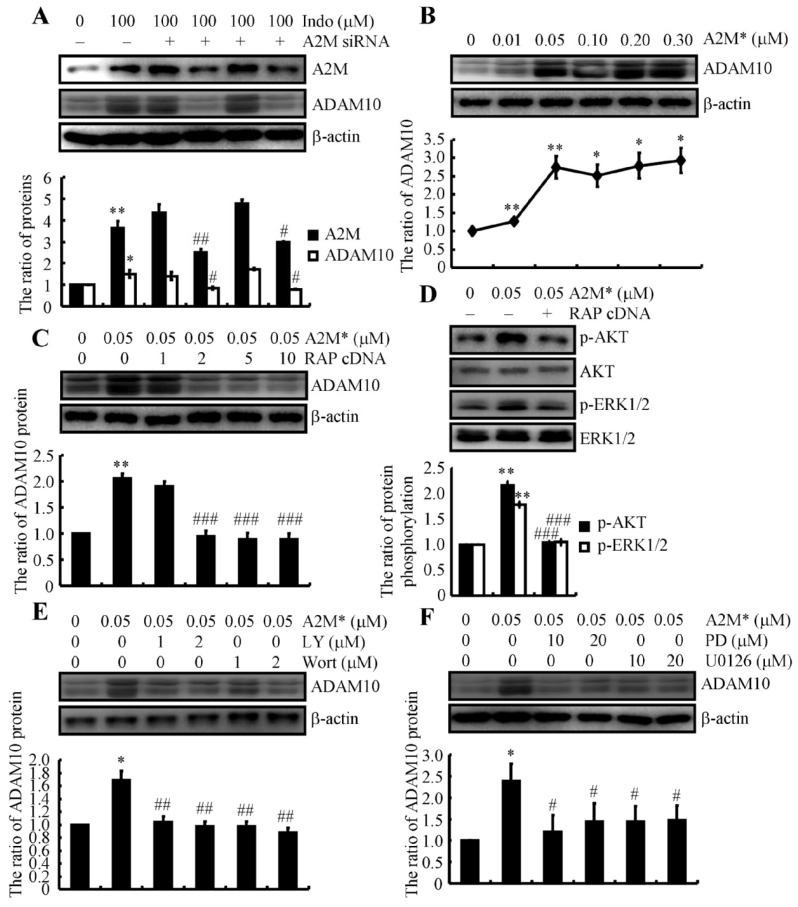
A2M induces ADAM10 expression via the PI3K and ERK1/2 pathways through a receptor-dependent mechanism. (**A**) N2a cells were treated with 100 μM indomethacin without or with A2M knockdown. (**B**) N2a cells were treated with the indicated concentrations of A2M* from 0.01 to 0.3 μM for 24 h. (**C**,**D**) N2a cells were treated with 0.05 μM A2M* without or with the ectopic expression of RAP. (**E**,**F**) N2a cells were treated with 0.05 μM A2M* in the absence or presence of the indicated concentrations of PI3-K inhibitor, LY294002 or wortmannin, and ERK1/2 inhibitor, PD98059 or U0126. Western blotting was performed to determine the expression of A2M and ADAM10. β-actin served as an internal control. The phosphorylation of AKT and ERK1/2 was also determined using Western blotting. Total AKT and ERK1/2 proteins served as internal controls. The optical density of the bands was analyzed using ImageJ software (v1.51, NIH, Bethesda, MD, USA). Data are presented as the means ± S.E. of independent experiments. * *p* < 0.05 and ** *p* < 0.01 compared to vehicle-treated controls. # *p* < 0.05, ## *p* < 0.01 and ### *p* < 0.001 compared to A2M*-treated cells.

By revealing the mechanisms of A2M induction, we continued to elucidate the important roles of A2M in AD. For this purpose, we knocked down the expression of A2M in indomethacin-treated N2a cells (Figure 5A). Upon knocking down A2M expression, we found that upregulation of ADAM10 was attenuated in indomethacin-treated N2a cells (Figure 5A). Reciprocally, we treated N2a cells with the indicated concentrations of the active form of A2M (from 0.01 to 0.30 μM) for 24 h. As shown in Figure 5B, treatment with 0.05 μM A2M* markedly increased the expression of the ADAM10 protein in N2a cells. Receptor-associated protein (RAP) is known to abolish the binding of almost all ligands to LRP1 [38]; therefore, we ectopically expressed RAP in A2M-treated N2a cells. Overexpression of RAP in N2a cells downregulated the expression of ADAM10 in A2M-stimulated cells (Figure 5C).

As downstream targets, the PI3-K and ERK1/2 pathways potentially contribute to mediating the effects of A2M on regulating ADAM10 expression [39]. We treated N2a cells with 0.05 μM of the active form of A2M in the absence or presence of ectopically expressed RAP to test this hypothesis. Western blots revealed increased phosphorylation of AKT and ERK1/2 without altering levels of the total AKT and ERK1/2 proteins in A2M*-stimulated N2a cells (Figure 5D). Moreover, overexpression of RAP blocked the effects of A2M* on AKT and ERK1/2 phosphorylation in N2a cells (Figure 5D). These results indicate the involvement of the PI3-K and ERK1/2 pathways in mediating the effects of A2M* on upregulating ADAM10 expression in N2a cells. We treated N2a cells with 0.05 μM A2M* in the absence or presence of a PI3-K inhibitor, LY294002 or wortmannin, or an ERK1/2 inhibitor, PD98059 or U0126, for 24 h to further validate the observations described above. The inhibition of either PI3-K or ERK1/2 attenuated the effects of A2M* on inducing ADAM10 expression (Figure 5E,F).

### 2.8. A2M Stimulates ADAM10 Expression via mTOR Signaling Pathways

**Figure 6:**
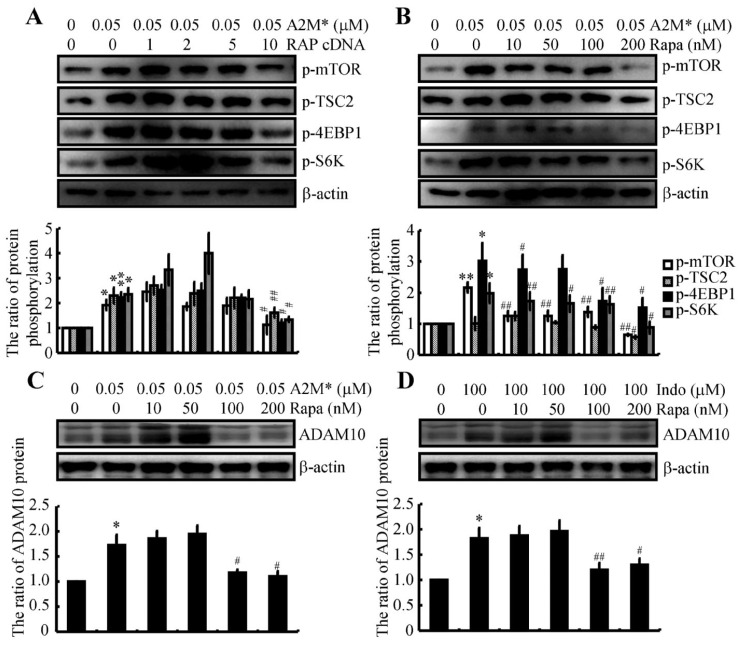
A2M induces ADAM10 expression via the mTOR pathway and a receptor-dependent mechanism. (**A**) N2a cells were treated with 0.05 μM A2M* without or with ectopic expression of RAP. (**B**,**C**) N2a cells were treated with 0.05 μM A2M* in the absence or presence of the indicated concentrations of the mTOR inhibitor rapamycin from 10 to 200 nM. (**D**) N2a cells were treated with 100 μM indomethacin in the absence or presence of the indicated concentrations of the mTOR inhibitor rapamycin ranging from 10 to 200 nM. Western blotting was used to determine phosphorylation of mTOR, TSC, 4EBP1 and S6K (**A**,**B**), as well as the expression of ADAM10 (**C**,**D**). β-actin served as an internal control. The optical density of the bands was analyzed using ImageJ software (v1.51, NIH, Bethesda, MD, USA). Data are presented as the means ± S.E. of independent experiments. * *p* < 0.05 and ** *p* < 0.01 compared to vehicle-treated controls. # *p* < 0.05 and ## *p* < 0.01 compared with A2M*- or indomethacin-treated cells.

In addition to the PI3-K and ERK1/2 pathways, mTOR is also activated by A2M following its recognition by a receptor on the cell surface [40]. Therefore, we assessed the involvement of the mTOR signaling pathway in mediating the effects of A2M on ADAM10 expression. For this purpose, RAP was ectopically expressed in N2a cells by transfecting RAP cDNA constructs. RAP overexpression in A2M*-treated N2a cells suppressed the phosphorylation of mTOR, 4EBP1 and S6K (Figure 6A). N2a cells were treated with A2M* in the absence or presence of the indicated concentrations of the mTOR inhibitor rapamycin to further examine the involvement of the mTOR in regulating ADAM10 expression. Treatment with 200 nM rapamycin markedly attenuated the effects of A2M* on activating mTOR signaling pathways, including the phosphorylation of mTOR, 4EBP1 and S6K, in N2a cells (Figure 6B). Consequently, the expression of ADAM10 was also inhibited by the addition of rapamycin (200 nM) to A2M* (0.05 μM)-stimulated N2a cells (Figure 6C). This observation was further reinforced by treatment with indomethacin in the absence or presence of the indicated concentrations of rapamycin (Figure 6D). Based on these observations, we can conclude that the mTOR signaling pathway seems to be involved in mediating these effects via mechanisms mediated by A2M* and its receptor.

### 2.9. A2M Mediates the Effects of Indomethacin on Suppressing the Aggregation of Aβ

**Figure 7:**
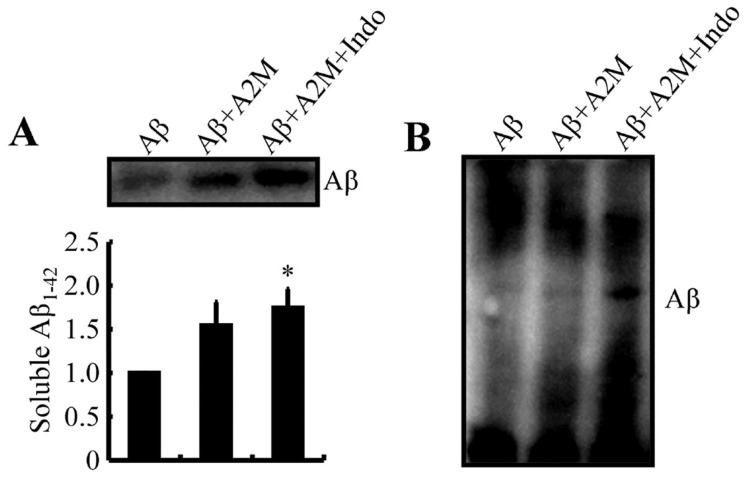
A2M mediates the effects of indomethacin on suppressing Aβ aggregation. (**A**,**B**) Levels of soluble and aggregated forms of Aβ incubated in the absence or presence of A2M and indomethacin were determined using Western blotting based on SDS-PAGE and native electrophoresis. Data are presented as the means ± S.E. of independent experiments. * *p* < 0.05 compared to vehicle-treated controls.

We incubated A2M with purified Aβ for 24 h to determine the role of A2M in Aβ aggregation. After centrifugation, supernatants were collected for Western blot analysis. As shown in Figure 7A, the level soluble Aβ was slightly increased upon the addition of the A2M but was significantly increased following the addition of indomethacin. The precipitated pellets were further loaded onto the gel, which was then probed with an Aβ antibody. Levels of high-molecular-weight aggregates of Aβ were slightly decreased, and levels of low-molecular-weight aggregates of Aβ were slightly increased after the addition of A2M (Figure 7B). This phenomenon was further corroborated by the addition of indomethacin to the mixture (Figure 7B). These observations indicate that indomethacin disrupts the aggregation of Aβ by activating A2M.

### 2.10. Indomethacin Blocks the Effects of Aβ by Inducing the Degradation of LRP1

**Figure 8:**
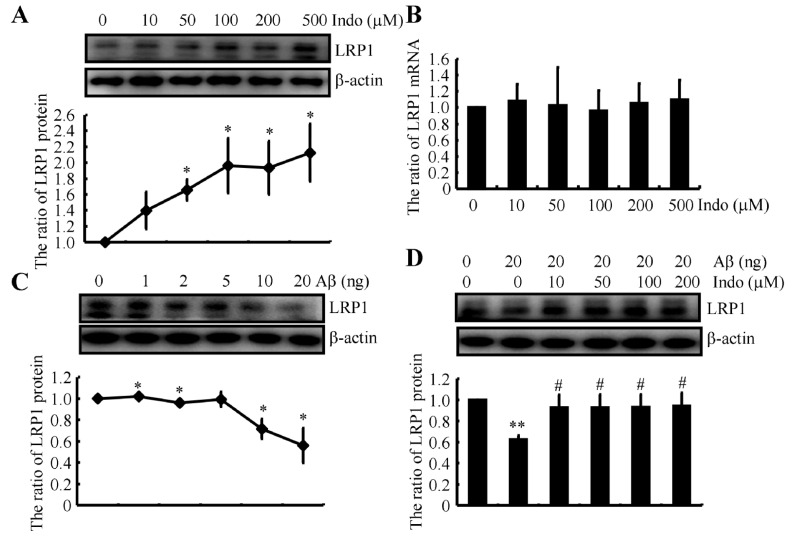
Indomethacin blocks the effects of Aβ on inducing LRP1 degradation. (**A**) N2a cells were treated with the indicated concentrations of indomethacin from 10 to 500 μM for 24 h. Western blotting was used to determine the level of LRP1 protein. β-actin served as an internal control. (**B**) The expression of the LRP1 mRNA was determined using qRT-PCR. GAPDH served as a housekeeping gene. (**C**) N2a cells were treated with the indicated concentrations of Aβ ranging from 1 to 20 ng for 24 h. (**D**) In some experiments, N2a cells were treated with 20 ng of Aβ in the absence or presence of the indicated concentrations of indomethacin, ranging from 10 to 200 μM for 24 h. Western blotting was employed to determine the expression of LRP1. β-actin served as an internal control. The optical density of the bands was analyzed using ImageJ software (v1.51, NIH, Bethesda, MD, USA). The data are presented as the means ± S.E. of independent experiments. * *p* < 0.05 and ** *p* < 0.01 compared with the vehicle-treated controls. # *p* < 0.05 compared with Aβ-treated groups.

As LRP is responsible for recognizing and clearing stable A2M-Aβ complexes [36], we next aimed to explore the roles of indomethacin in LRP activation. In response to treatment with indomethacin, levels of the LRP1 protein were increased in a dose-dependent manner in N2a cells (Figure 8A). However, the expression of the LRP1 mRNA was not altered by treatment with indomethacin in the same cells (Figure 8B). These observations indicate the protective effects of indomethacin on the degradation of LRP1. In Aβ-treated N2a cells, Aβ decreased the levels of the LRP1 protein (Figure 8C). More interestingly, indomethacin blocked the effects of Aβ on reducing the levels of the LRP1 protein in N2a cells (Figure 8D). Based on these findings, we can conclude that indomethacin likely protects LRP1 from degradation in subjects with AD, which contributes to the efflux of Aβ from the brain.

## 3. Discussion

A2M is strictly regulated under physiological or pathological conditions. Regarding the A2M gene, Matthjis et al. first identified a polymorphism with a deletion near the 5′ splice site of exon 18 (A2M-2) in 1991 [41]. In case–control studies, the allele frequency of A2M-2 is higher in individuals with AD than in healthy populations, suggesting a potential relationship between A2M-2 and the risk of AD [42,43]. In addition, A2M-2 does not alter the expression of the A2M mRNA and protein [44]. Subsequently, a single amino acid substitution at position 1000 (1000 V/I) was identified as another AD-associated polymorphism [45], and A2M-2 is potentially associated with the 1000 V/I polymorphism [46]. More interestingly, both of these polymorphisms are associated with the deposition of Aβ [46]. However, these associations should be interpreted with caution due to the case-based study design [42,43,45], as the results were not consistently confirmed in subsequent family-based studies [47,48]. Interestingly, these controversies may be reconciled by the fact that A2M-2 is a greater risk factor for very late onset of AD in case–control studies of subjects over 80 years old [46,49].

In addition to genetic polymorphisms, the expression of the A2M protein was first identified to be associated with AD in immunohistochemical studies [50,51]. Specifically, elevated levels of A2M were observed in large hippocampal neurons and APs of patients with AD. However, subsequent studies suggested that A2M localizes specifically to neuritic APs but not to diffuse APs [52]. In a separate investigation, A2M levels were increased in the temporal cortices of patients with AD compared to control subjects [53]. Based on these results, we extended these prior findings to reveal that indomethacin treatment induces the colocalization of A2M and APs in the brains of APP/PS1 Tg mice (Figure 2D).

Given the association of A2M with AD, an increase in A2M expression should exert a biological effect on AD. Treatment with the active form of A2M revealed the involvement of the PI3-K, ERK1/2 and mTOR pathways in mediating the effects of A2M* on stimulating ADAM10 expression in N2a cells (Figure 5 and Figure 6). Consistent with our observations, the PI3-K, ERK1/2 and mTOR pathways are activated by the active form of A2M [39,40]. In addition, activation of the PI3-K/AKT pathway results in upregulated expression of ADAM10 in RXFP1-expressing cells [54]. Although we did not observe direct evidence showing the regulatory relationship among A2M, ERK1/2 and ADAM10, a previous report showed that ERK1/2 mediates the effects of A2M on upregulating MMP-9 expression in macrophage-derived cell lines [55]. In addition, indomethacin increases the production of sAPPα [56]. Moreover, we extended previous findings [40] by showing that A2M and its receptors induce ADAM10 expression by activating mTOR signaling pathways in N2a cells (Figure 6).

As a panprotease inhibitor, A2M activation by indomethacin disrupts the formation of Aβ aggregates (Figure 7A,B). Consistent with our results, A2M disrupts the formation of APs in cultured fetal rat cortical neurons [37]. As an extracellular chaperone, A2M inhibits the formation of APs through two distinct mechanisms: (1) by trapping proteases that remain able to degrade polypeptide chains and (2) by a chaperone action that prevents misfolded clients from continuing along the Aβ formion pathway [57]. Indeed, A2M specifically binds Aβ, mediating its endocytosis via cell surface low-density lipoprotein receptor-related protein (LRP) [36], which facilitates the clearance of Aβ deposits [37]. Specifically, the interaction between Aβ and LRP mediates the efflux of Aβ isoforms [58]. Similarly, A2M mediated the interaction between Aβ and LRP, resulting in the efflux of Aβ from the brains of APP/PS1 Tg mice.

Based on these results, we speculate that a marked increase in A2M expression might be a beneficial treatment for AD. We tested this hypothesis and identified indomethacin as a novel stimulator that upregulates the expression of A2M in the brains of APP/PS1 Tg mice (Figure 2A,B). Although no report has suggested the regulatory effects of indomethacin on A2M, indomethacin has been widely accepted as a nonspecific inhibitor of COX [26]. As COX-1 is constitutively expressed in many cell types and is presumed to be responsible for the synthesis of housekeeping PGs, we determined the effects of indomethacin on the activity of the inducible enzyme COX-2. Treatment with indomethacin suppressed not only the expression of COX-2 but also its downstream metabolic enzyme, L-PGDS (Figure 3). These observations indicate the involvement of the L-PGDS metabolic product PGD_2_ in the expression of A2M in neurons. Therefore, we treated N2a cells with PGD_2_ and found that PGD_2_ downregulates the expression of A2M via its receptor, CRTH2 (Figure 4). These new findings fill the gaps between indomethacin and A2M, ameliorating the phenotype of APP/PS1 Tg mice.

In addition to A2M, LRP has been reported to be associated with AD based on genetic and biochemical evidence. Genetic polymorphisms of LRP are associated with late-onset familial AD [59,60]. Furthermore, reduced levels of LRP in individuals with AD are associated with brain Aβ accumulation [60,61,62]. Ablation of LRP suppresses rapid Aβ brain capillary clearance and efflux at the BBB [58]. Consistently, Aβ robustly accumulates in the brains of Tg 2576 mice that produce mutant low-clearance LRP [63]. More specifically, endothelial LRP1 transports Aβ_1–42_ across the BBB [64]. In addition, the function of LRP1 in clearing Aβ is not limited to brain vascular cells and includes neuronal cells [65]. By binding to secreted APP, LRP affects its degradation [61] and processing [66], leading to Aβ production [67]. In addition, overexpression of functional LRP in neurons of AD mice results in the generation of soluble Aβ in the brain [68]. By producing soluble Aβ, LRP also disrupts the aggregation and deposition of Aβ. Based on these observations, we extended previous studies by showing that Aβ induces the degradation of LRP1, which was blocked by the addition of indomethacin in neurons (Figure 8A–D).

In addition to its effects on A2M, indomethacin was reported to be an effective treatment for AD as early as 1993 in a small randomized controlled trial [21]. Similarly, indomethacin also decreases the accumulation of Aβ in the brains of Tg2576 mice [25]. Moreover, as an anti-inflammatory agent, indomethacin reverses the activity of microglia surrounding the Aβ deposits after an infusion into the lateral ventricles of rats [18]. We extended these previous studies and revealed that indomethacin suppresses Aβ production by increasing ADAM10 expression in the brains of APP/PS1 Tg mice (Figure 1D,E). In addition, indomethacin treatment decreased the deposition of Aβ in APs (Figure 1G). Therefore, indomethacin improved cognitive decline in APP/PS1 Tg mice by suppressing the production and deposition of Aβ and inducing Aβ efflux through a mechanism mediated by A2M-induced LRP1 activation.

As the inhibitors of COXs, long-term treatment with NSAIDs in AD patients has shown its effects on reducing the risk of the disease by 50% in several large-scale epidemiological and observational studies [69,70,71]. To the mechanisms, NSAIDs primarily exert their neuroprotective effects via inhibiting the activities of COXs, leading to a decrease in the synthesis of PGE_2_, which accelerates the clearance of Aβ. In the study of AD animal models, NSAIDs can improve the behavioral and pathological defects [72,73]. However, the results of NSAIDs in clinical trials are not ideal. Biomarker studies have shown that Aβ has deposited in APs before the appearance of symptoms of AD cognitive impairment [74,75]. Once Aβ is deposited in APs, NSAIDs could not reverse the process and may even be harmful since they inhibit the activities of microglia from clearing Aβ. This may be the reason why NSAID has limited therapeutic effect on AD patients. At present, studies on NSAIDs mainly focus on their potential biological effects on AD. A large amount of evidence shows that NSAIDs may reduce the risk of AD and delay the development and progression of the disease. This is also the case in AD patients in that long-term treatment with NSAIDs decreases the rate of mortality [76,77].

## 4. Materials and Methods

### 4.1. Reagents

Indomethacin was purchased from Chengdu Aikeda Chemical Reagent Co., Ltd. (Chengdu, Sichuan, China). Bay-u3405 was obtained from Adooq Bioscience (Nanjing, Jiangsu, China). Human Aβ_1–42_ was purchased from ChinaPeptides (Suzhou, Jiangsu, China). PGD_2_ was purchased from Sigma-Aldrich Corp. (St. Louis, MO, USA). Antibodies against β-actin, LRP1, Aβ, ADAM10, p-AKT, AKT, p-ERK1/2, ERK1/2, p-mTOR, p-TSC2, p-4EBP1 and p-S6K were obtained from Cell Signaling Technology, Inc. (Danvers, MA, USA). Antibodies specific for COX-2 were purchased from Abcam (Shanghai, China). Antibodies specific for A2M, L-PGDS and CRTH2 were purchased from Santa Cruz Biotechnology (Shanghai, China). All reagents used in the qRT-PCR and SDS-PAGE experiments were purchased from Bio-Rad Laboratories (Shanghai, China). All other reagents were purchased from Sigma-Aldrich Corp. (St. Louis, MO, USA) unless specified otherwise.

### 4.2. Cell Culture and Treatment

Mouse neuroblastoma 2a (N2a) cells were grown (37 °C and 5% CO_2_) on 6 cm tissue culture dishes (1 × 10^6^ cells per dish) in an appropriate medium. In a separate set of experiments, the cells were grown in serum-free medium for an additional 12 h before incubation with the indicated concentrations of different reagents. For indomethacin, the cells were treated with the drug at the concentrations of 0, 10, 50, 100, 200 or 500 μM for 24 h. For PGD_2_ treatment, 0, 1, 2, 5, 10 or 20 μM of the drug was used to treat N2a cells. To block the CRTH2, N2a cells were treated at the indicated concentrations of Bay-u3405, including 0, 1, 2, 5 or 10 μM in the presence of 10 μM PGD_2_. To determine the effects of A2M* on the cells, N2a cells were treated with 0.05 μM A2M* in the absence or presence of the mTOR inhibitor, rapamycin (0, 10, 50, 100 or 200 nM) or transfection of the RAP cDNA. In the experiments of LRP1 degradation, the cells were treated with 0 to 20 ng of Aβ in the absence or presence of indicated concentrations of indomethacin ranging from 0 to 200 μM for 24 h. The cells were then collected and lysed with RIPA buffer for Western blot analyses.

### 4.3. Transfection

Cells were transfected with 1.6 μg of the RAP cDNA constructs using Lipofectamine 2000 (Thermo Fisher Scientific, Shanghai, China) to ectopically express RAP. Briefly, 1 × 10^6^ cells were seeded in 6 cm tissue culture dishes. After attachment, the cells were incubated with serum-deprived medium without antibiotics for 12 h before transfection with the cDNA. The cDNA templates were diluted in 50 μL of Opti-MEM and incubated at room temperature for 5 min. In a separate tube, 1 μL Lipofectamine 2000 was also incubated in 50 μL of Opti-MEM for 5 min, after which it was mixed with the siRNA or cDNA solution. After 25 min, the mixture was added to N2a cells. Transfected cells were allowed to recover for at least 12 h in growth medium and then incubated overnight with serum-free medium before adding A2M* or *hAβ* to the cultured cells.

### 4.4. Transgenic Mice

C57BL/6 mice were purchased from Liaoning Changsheng Biotechnology Co., Ltd. (Benxi, Liaoning, China). APP/PS1 Tg mice [B6C3-Tg (APPswe, PSEN1dE9) 85Dbo/J (stock number: 004462)] were obtained from Jackson Laboratory (Bar Harbor, ME, USA). Genotyping was performed 3–4 weeks after birth. Briefly, 1 mm of mouse tails were placed in 150 μL of 50 mM NaOH and heated to 95 °C for 30 min. The mixture was then cooled to room temperature and vortexed, and 12.5 μL of Tris–HCl was added to adjust the pH to 8.0. The extracted DNA was centrifuged at 1 × 10^4^ rpm for 2 min, and 2 μL of the supernatant was removed for PCR amplification. The forward and reverse primers for genotyping were 5′-AATAGAGAACGGCAGGAGCA-3′ and 5′-GCCATGAGG GCACTAATCAT-3′, respectively. The forward and reverse primers for the internal control were 5′-CTAGGCCACAGAATTGAAAGATCT-3′ and 5′-GTAGGTGGA AATTCTAGCATCATCC-3′, respectively. The reaction mixtures were incubated at 94 °C for 3 min and then subjected to 35 PCR cycles with the following temperature profile: 94 °C for 45 s, 60 °C for 30 s and 72 °C for 5 min. The DNA products of PCR were analyzed by electrophoresis on 2% agarose gels. Bands of 608 and 324 bp were expected for the transgenic mice and internal controls, respectively. Mice were housed in groups of six per cage in a controlled environment at standard room temperature and relative humidity on a 12 h light–dark cycle and had free access to food and water. For each in vivo study, six mice in each group were studied. In selected experiments, at 6-month-old mice were intragastric administered indomethacin (3 mg/kg/d) for 3 months before assessing their learning ability using the Morris water maze test or the nest construction assay.

### 4.5. Morris Water Maze

Mice were trained and tested in a Morris water maze as previously described [78]. Briefly, mice were pretrained in a circular water maze with a visible platform for 2 d. The platform was then submerged inside the maze, with the deck located 0.5 cm below the surface of the water for subsequent experiments. Milk was added to the water to hide the platform from sight. Mice were placed inside the maze and allowed to swim freely until they found the hidden platform. The entire experiment lasted for 7 d. Mice were left in the maze for a maximum of 60 s and allowed to find the platform. The learning sessions were repeated in 4 trials per day with an interval of 1 h between each session. Spatial learning scores (the latency period necessary to find and climb onto the hidden platform and the length of the path to the platform) were recorded. On the last day, the platform was removed, and the number of times that the mice passed through the memorized region was recorded for a period of 60 s. Finally, recorded data were statistically analyzed using a computer program (ZH0065; Zhenghua Bioequipment, Yuanyang City, Henan, China).

### 4.6. Nest Construction

Nest construction was tested and analyzed as previously described [78]. Briefly, mice were housed in a cage with corncob bedding for 1 week before the nest construction test. Two hours before the onset of the dark phase of the light/dark cycle, 8 pieces of paper (5 × 5 cm^2^) were introduced into the home cage to create conditions for nesting. Nests were scored the next morning using a 4-point system: 1, no biting/tearing of paper, with random dispersion of the paper; 2, no biting/tearing of paper, with gathering of the papers in a corner/side of the cage; 3, moderate biting/tearing of paper, with gathering of the papers in a corner/side of the cage; and 4, extensive biting/tearing of paper, with gathering of the papers in a corner/side of the cage.

### 4.7. Stereotaxic Injection

PGD_2_ or vehicle (PBS) was injected (i.c.v) into mice as previously described [78]. Briefly, stereotaxic injectors were placed at the following coordinates relative to the bregma: mediolateral, −1.0 mm; anteroposterior, −0.22 mm; and dorsoventral, −2.8 mm. Five microliters of chemical reagents were slowly injected into the ventricles of APP/PS1 Tg mice. The injector was removed gently after the injection. Twenty-four hours after the injection, mice were anesthetized and subjected to perfusion as previously described [78,79].

### 4.8. Intragastric Administration

APP/PS1 Tg mice were intragastrically administered the indicated concentration of indomethacin by gavage. Briefly, a suitable gavage needle was selected and fixed on a 1 mL syringe. The mice were then prepared by aligning the head, neck and body. The gavage needle was introduced into the corner of the mouth, pressed against the tongue, pushed inwardly against the upper jaw and ultimately introduced into the esophagus. The gavage needle was removed gently after the injection.

### 4.9. Collection of Brains

Mouse brains were collected from WT or APP/PS1 Tg mice. Each mouse was sacrificed by CO_2_ inhalation in an enclosed space and then quickly fixed on an operating table. The heart was exposed by opening the chest, and a perfusion needle was inserted into the left ventricle. The perfusion needle was fixed with hemostatic forceps, and the right atrial appendage was cut with scissors. Perfusion was performed with physiological saline and was stopped when the liver became white and a clear solution flowed from the right atrium. The brains were collected on ice and separated equally into two parts. One part was submerged in 4% paraformaldehyde; the other part was stored at −80 °C until protein extraction was performed.

### 4.10. Immunofluorescence Staining

Brain tissues were collected from APP/PS1 transgenic mice. Serial sections (10 μm thick) were cut using a cryostat (Leica, CM1850, Buffalo Grove, IL, USA). Slides were rehydrated in a graded series of ethanol and submerged in 3% hydrogen peroxide to eliminate endogenous peroxidase activity. Colocalization of A2M and Aβ was determined using an immunofluorescence staining kit according to the manufacturer’s instructions (MXB Biotechnologies, Fuzhou, Fujian, China). Briefly, frozen sections of mouse brains were treated with 5% BSA for 1 h and then incubated with a rabbit anti-A2M antibody together with a mouse anti-Aβ overnight at 4 °C. After washes, sections were incubated with Alexa Fluor 555- or 488-conjugated secondary antibodies for 1 h at room temperature. After thorough rinsing, stained sections were dehydrated, cleared and mounted with a fluorescent sealing agent. The sections were examined, and images were obtained using a Leica microscope (Leica, CM1850, Buffalo Grove, IL, USA).

### 4.11. Tissue Embedding

The brains were immobilized in 4% paraformaldehyde for 48 h, soaked in 70% ethanol for 5 h, and soaked in 80% ethanol overnight at 4 °C. For dehydration, the tissues were soaked sequentially in 90% ethanol for 45 min, 95% ethanol for 30 min (twice), 100% ethanol for 30 min (twice), and 100% ethanol for 45 min. The tissues were then placed in xylene for 20 min and a xylene:soft wax mixture for 45 min before being embedded in soft wax for 50 min and hard wax for 50 min. Throughout the process, the organization remained intact; the clearing time was carefully controlled, as an excessive clearing duration can easily disrupt tissue organization. After tissue embedding, serial sections (5 μm thick) were cut using a paraffin slicer (Leica, RM2235, Buffalo Grove, IL, USA), and the sections were used for morphological determination.

### 4.12. Immunohistochemistry (IHC)

Slides were first dewaxed and rehydrated through a series of xylene and ethanol washes, as follows: 100% xylene for 20 min, 100% ethanol II for 10 min, 100% ethanol I for 10 min, 95% ethanol II for 10 min, 95% ethanol I for 5 min, 90% ethanol for 5 min, 80% ethanol for 5 min, 70% ethanol for 5 min and deionized water for 5 min. The slides were boiled in a solution of sodium citrate for 20 min for antigen retrieval before cooling to room temperature. The tissues were then submerged in 30% hydrogen peroxide to eliminate endogenous peroxidase activity and blocked with goat serum for 45 min. The localization of Aβ was determined using an immunohistochemical staining kit (Fuzhou, Fujian, China) according to the manufacturer’s instructions. Briefly, the tissues were incubated with primary antibodies at 4 °C overnight. The next day, the primary antibodies were removed, and the tissues were washed three times with phosphate buffer (PB) for 5 min. Tissues were then incubated with secondary antibody at room temperature for 1 h and with streptomycin antibiotin peroxidase for 1 h. After three rinses with PB three times for 5 min, the slides were visualized with DAB, and the reaction was stopped with deionized water. The slides were finally dehydrated in 70% ethanol for 5 min, 80% ethanol for 5 min, 90% ethanol for 5 min, and 95% ethanol I for 5 min, 95% ethanol II for 10 min and cleared in 100% xylene for 20 min. After air-drying in a fume hood, the slides were mounted with neutral resin before being observed under a microscope.

### 4.13. Western Blot Analysis

Tissues or cells were washed with PBS (-) and lysed in 500 μL of RIPA buffer containing a protease inhibitor cocktail (Pierce Chemical Company, Shanghai, China). The protein content in the cell lysates was determined using a bicinchoninic acid (BCA) protein assay reagent (Pierce Chemical Company, Shanghai, China). Protein samples were mixed with loading buffer and boiled for 5 min at 100 °C. Aliquots of total cell lysates were subjected to SDS-PAGE, transferred to a nitrocellulose membrane and probed with a panel of specific antibodies. Each membrane was probed with only one antibody. β-actin was used as a loading control. All Western blot hybridizations were performed at least in triplicate, and a different cell preparation was used each time.

### 4.14. RNA Extraction

Total RNA was extracted using RNA extraction kits (Promega Corp., Beijing, China). Briefly, the culture medium was discarded before adding 0.5 mL of TRIzol to lyse the cells, which were then scraped down and transferred to Eppendorf tubes. Then, 0.25 mL of chloroform was added to the tube, which was then vortexed and centrifuged at 1.5 × 10^4^ rpm for 5–7 min. The supernatant was collected and mixed with 2 mL of 70% ethanol, which was then loaded on the RNA extraction columns. The columns were sequentially washed with 0.5 mL of Buffer I and Buffer II by centrifugation at 1 × 10^4^ rpm for 1 min. Finally, 40 μL of deionized water was added to the membrane of columns to elute the RNA, which was stored at −80 °C until qRT-PCR.

### 4.15. Reverse Transcription

Between 2 and 5 μg of RNA was reverse-transcribed to cDNAs using reverse transcription kits (Promega Corp., Beijing, China). Briefly, RNA was mixed with 4 μL of 5 × buffer, 1.5 μL of MgCl_2_, 1 μL of dNTPs, 0.5 μL of nuclease inhibitor, 1 μL of oligo dT primers, 1 μL of random primer and 1 μL of reverse transcriptase. The reaction mixtures were incubated at 25 °C for 5 min, 42 °C for 1 h and 70 °C for 15 min.

### 4.16. qPCR

qPCR assays were performed with the MiniOpticon Real-Time PCR detection system (Bio-Rad Laboratories, Shanghai, China) using total RNA and the GoTaq One-step Real-Time PCR kit with SYBR green (Promega, Beijing, China). Forward and reverse primers for mouse LRP1 were 5′-ATGAGCACAGTTGTCTGGGG-3′ and 5′-AGGAGCCATCTGTGTTGGTG-3′, respectively. The gene expression levels were normalized to GAPDH, whose forward and reverse primers were 5′-GAGAGTGTTTCCTCGTCCCG-3′ and 5′-ACTGTGCCGTTGAATTTGCC-3′, respectively. Reaction mixtures were incubated at 50 °C for 15 min followed by 95 °C for 5 min, and then 35 PCR cycles were performed with the following temperature profile: 95 °C for 15 s, 58 °C for 30 s, 68 °C for 1 min and 77 °C for 20 s. Data were collected at a specific step (77 °C for 20 s) to remove possible fluorescent contributions from primer dimers. Gene expression values were normalized to GAPDH.

### 4.17. Measurement of Aβ_1–42_ Concentrations

The concentrations of Aβ_1–42_ were determined as previously described [80]. Briefly, the Aβ_1–42_ kit is a solid phase sandwich enzyme-linked immunosorbent assay (ELISA) kit that was purchased from Thermo Fisher Scientific/Life Technologies (Shanghai, China). A monoclonal antibody against the NH_2_-terminus of Aβ was used to coat the wells of the microtiter strips provided. During the first incubation, standards with a known Aβ_1–42_ content, controls and unknown samples were pipetted into the wells and coincubated with a rabbit antibody against the COOH terminus of the Aβ_1–42_ sequence. This COOH-terminal sequence is created upon cleavage of the analyzed precursor. After washing, the bound rabbit antibody was detected by incubating the strip with a horseradish peroxidase-labeled anti-rabbit antibody. After a second incubation and washing step to remove all unbound enzymes, a substrate solution was added, which was acted upon by the bound enzyme to produce a colored product. The intensity of this colored product is directly proportional to the concentration of human Aβ_1–42_ present in the original specimen.

### 4.18. Brain Tissue Extracts

The brains were removed and isolated from the mice as previously described unless stated otherwise [78,79]. Tissues were then homogenized in RIPA buffer, vortexed, sonicated for 3.5 s and centrifuged at 3000× *g* for 5 min. The supernatant was then aliquoted and frozen at −80 °C.

### 4.19. Semidenaturing Detergent Agarose Gel Electrophoresis

Semidenaturing detergent agarose gel electrophoresis (SDD-AGE) was performed as previously described [81]. Gels containing a 1.5% agarose solution in 1× Tris-acetate buffer (TAE) and 0.1% SDS were prepared. Gels were subjected to electrophoresis at a low voltage (3 V/cm gel length) until the dye front reached ~1 cm from the end of the gel. Gels were then transferred to nitrocellulose membranes for a minimum of 3 h. After transfer, membranes were processed using standard Western blot techniques to visualize the aggregated form of Aβ.

### 4.20. Aβ Aggregation Assay

First, 100 ng of Aβ_1–42_ was incubated with 50 nM A2M in the absence or presence of 100 μM indomethacin in PBS containing Triton X-100 in a total incubation volume of 40 μL at 37 °C for 2 h. Monomeric Aβ was separated from Aβ aggregates using a 10,000 KDa cutoff filter. Monomeric and aggregated Aβ were subjected to SDS-PAGE or SDD-AGE electrophoresis, respectively. Proteins were then transferred to nitrocellulose membranes and visualized using an Aβ-specific antibody.

### 4.21. A2M* Preparation

A2M from human plasma was obtained from Sigma-Aldrich Corp. (St. Louis, MO, USA). Transformed methylamine (MA)-A2M* was generated as previously described with minor modifications [82]. Briefly, A2M was reacted with 200 mM methylamine-HCl in 50 mM Tris-HCl, pH 8.2 at 25 °C. Excess methylamine was removed by dialysis against 20 mM sodium phosphate (150 mM NaCl, pH 7.4; PBS).

### 4.22. Brain Retention and Efflux of Aβ

First, *hAβ* (2 μg/5 μL) was injected (i.c.v.) into the brains of WT mice, which were orally pre-administered indomethacin (3 mg/kg) for 2 h. Then, brains were collected at the indicated time points without perfusion. The brains were homogenized by sonication on ice. The supernatant was collected by centrifugation at 1.5 × 10^4^ rpm for 10 min. The remaining *hAβ* in the supernatant was analyzed using an ELISA. Brain retention and efflux of *hAβ* were calculated using the following equations:

$$\mathrm{Brain}\;\mathrm{retention}\;\mathrm{of}\;hA\beta \;(\%)\;=\;\frac{{Remaining\;hA\beta }_{Indicated\;Time}}{Total\;injected\;hA\beta }\;\times 100$$

Brain efflux of *hAβ* = *Log*[ 100 − *Brain retainment of hAβ* (%)]

### 4.23. Animal Committee

All animals were handled according to the Guide for the Care and Use of Medical Laboratory Animals (Ministry of Health, People’s Republic of China, 1998). All experimental protocols were approved by the Laboratory Ethics Committees of the College of Life and Health Sciences of Northeastern University (NEU-EC-2021A014S).

### 4.24. Statistical Analysis

All data are presented as the means ± S.E. Differences among means were analyzed using analysis of variance (ANOVA). The Newman–Keuls post hoc test was used to assess pairwise comparisons between means. Differences between means were analyzed using a two-tailed Student’s *t*-test [29]. In all analyses, the null hypothesis was rejected at the 0.05 level. Statistical analyses were performed using the Prism Stat program (GraphPad Software, Inc., San Diego, CA, USA).

## 5. Conclusions

Our results reveal the mechanisms by which indomethacin decreases the production and deposition of Aβ, which results in the amelioration of cognitive decline in APP/PS1 Tg mice. Our novel findings are that indomethacin treatment concurrently increases the expression of A2M and decreases the degradation of LRP1, leading to the efflux of Aβ from the brains of AD animals. Mechanistically, indomethacin upregulates the expression of A2M by suppressing COX-2 and L-PGDS expression and the synthesis of PGD_2_ and its receptor, CRTH2. By inducing A2M expression, the production and aggregation of Aβ were inhibited, which resulted in the amelioration of cognitive decline in APP/PS1 Tg mice. Based on these findings, it can be concluded that indomethacin is beneficial to AD. Therefore, indomethacin might be used as an adjuvant drug to prevent the onset of AD or delay the progression of the disease. Since we did not observe the effects of indomethacin on reversing the pathological characteristics of AD, indomethacin might not be used as a therapeutic drug for treating AD, especially at the late stage of the disease.

## Data Availability

The data presented in this study are available upon request from the corresponding author.

## Abbreviations

NSAID	nonsteroidal anti-inflammatory drug
AD	Alzheimer’s disease
A2M	α2-macroglobin
COX-2	cyclooxygenase-2
L-PGDS	lipocalin-type prostaglandin D synthase
L-PGDS	lipocalin-type prostaglandin D synthase
PG	prostaglandin
CRTH2	PGD_2_ receptor 2
Aβ	β-amyloid protein
LRP1	low-density lipoprotein receptor-related protein 1
Tg	transgenic
APs	β-amyloid plaques
NFTs	neurofibrillary tangles
iNOS	inducible nitric oxide synthase
WT	wild type
N2a	neuroblastoma 2a

## References

[1] Ott A., Breteler M.M.B., Harskamp F.V., Claus J.J., Cammen T.J.M.V.D., Grobbee D.E., Hofman A. (1995). Prevalence of Alzheimer’s disease and vascular dementia: Association with education. The Rotterdam study. BMJ.

[2] Kent S.A., Spires-Jones T.L., Durrant C.S. (2020). The physiological roles of tau and Aβ: Implications for Alzheimer’s disease pathology and therapeutics. Acta Neuropathol..

[3] Guan P.P., Wang P. (2019). Integrated communications between cyclooxygenase-2 and Alzheimer’s disease. FASEB J..

[4] Wang P., Wang Z.Y. (2017). Metal ions influx is a double edged sword for the pathogenesis of Alzheimer’s disease. Ageing Res. Rev..

[5] Golde T.E. (2002). Inflammation takes on Alzheimer disease. Nat. Med..

[6] McGeer E.G., McGeer P.L. (2010). Neuroinflammation in Alzheimer’s disease and mild cognitive impairment: A field in its infancy. J. Alzheimers Dis..

[7] Szekely C.A., Thorne J.E., Zandi P.P., Ek M., Messias E., Breitner J.C., Goodman S.N. (2004). Nonsteroidal anti-inflammatory drugs for the prevention of Alzheimer’s disease: A systematic review. Neuroepidemiology.

[8] Thomas T., Nadackal T.G., Thomas K. (2001). Aspirin and non-steroidal anti-inflammatory drugs inhibit amyloid-β aggregation. Neuroreport.

[9] Eriksen J.L., Sagi S.A., Smith T.E., Weggen S., Das P., McLendon D.C., Ozols V., Jessing K., Zavitz K., Koo E. (2003). AIDs and enantiomers of flurbiprofen target β 3-secretase and lower Aβ242 in vivo. J. Clin. Investig..

[10] Morihara T., Chu T., Ubeda O., Beech W., Cole G.M. (2010). Selective inhibition of Aβ242 production by NSAID R-enantiomers. J. Neurochem..

[11] Weggen S., Eriksen J.L., Das P., Sagi S.A., Wang R., Pietrzik C.U., Findlay K.A., Smith T.E., Murphy M.P., Bulter T. (2001). A subset of NSAIDs lower amyloidogenic Aβ242 independently of cyclooxygenase activity. Nature.

[12] Jantzen P.T., Connor K.E., Dicarlo G., Wenk G.L., Wallace J.L., Rojiani A.M., Coppola D., Morgan D., Gordon M.N. (2002). Microglial Activation and β-Amyloid Deposit Reduction Caused by a Nitric Oxide-Releasing Nonsteroidal Anti-Inflammatory Drug in Amyloid Precursor Protein Plus Presenilin-1 Transgenic Mice. J. Neurosci..

[13] Quinn J., Montine T., Morrow J., Woodward W.R., Eckenstein F. (2003). Inflammation and cerebral amyloidosis are disconnected in an animal model of Alzheimer’s disease. J. Neuroimmunol..

[14] Kukar T., Prescott S., Eriksen J.L., Holloway V., Murphy M.P., Koo E., Golde T., Nicolle M. (2007). Chronic administration of R-flurbiprofen attenuates learning impairments in transgenic amyloid precursor protein mice. BMC Neurosci..

[15] Pratico D., Yao Y., Uryu K., Yang H., Trojanowsi J.Q., Lee V.-M.Y. (2003). Opposite Effects of Indomethacin and Nimesulide on Levels and Amyloid Deposition in a Transgenic Mouse Model of Alzheimer Amyloidosis.

[16] Sanjay Kumar Rao A., Chittaranjan Andrade B., Kishore Reddy B., Madappa B.K.N., Shivashanmugam Thyagarajan B., Suresh Chandra C. (2002). Memory protective effect of indomethacin against electroconvulsive shock-induced retrograde amnesia in rats. Biol. Psychiatry.

[17] Wang J., Cheng X., Zhang X., Liu G., Wang Y., Zhou W., Zhang Y. (2019). A combination of indomethacin and atorvastatin ameliorates cognitive and pathological deterioration in PrP-hAβPPswe/PS1△E9 transgenic mice. J. Neuroimmunol..

[18] Netland E.E., Newton J.L., Majocha R.E., Tate B.A. (1998). Indomethacin reverses the microglial response to amyloid beta-protein. Neurobiol. Aging.

[19] Ogawa O., Umegaki H., Sumi D., Hayashi T., Nakamura A., Thakur N.K., Yoshimura J., Endo H., Iguchi A. (2000). Inhibition of inducible nitric oxide synthase gene expression by indomethacin or ibuprofen in beta-amyloid protein-stimulated J774 cells. Eur. J. Pharm..

[20] Sung S., Yang H., Uryu K., Lee E.B., Zhao L., Shineman D., Trojanowski J.Q., Lee V.M., Pratico D. (2004). Modulation of nuclear factor-kappa B activity by indomethacin influences A beta levels but not A beta precursor protein metabolism in a model of Alzheimer’s disease. Am. J. Pathol..

[21] Rogers J., Kirby L.C., Hempelman S.R., Berry D.L., McGeer P.L., Kaszniak A.W., Zalinski J., Cofield M., Mansukhani L., Willson P. (1993). Clinical trial of indomethacin in Alzheimer’s disease. Neurology.

[22] De Jong D., Jansen R., Hoefnagels W., Jellesma-Eggenkamp M., Verbeek M., Borm G., Kremer B. (2008). No effect of one-year treatment with indomethacin on Alzheimer’s disease progression: A randomized controlled trial. PLoS ONE.

[23] Vlad S.C., Miller D.R., Kowall N.W., Felson D.T. (2008). Protective effects of NSAIDs on the development of Alzheimer disease. Neurology.

[24] Varvel N.H., Bhaskar K., Kounnas M.Z., Wagner S.L., Yang Y., Lamb B.T., Herrup K. (2009). NSAIDs prevent, but do not reverse, neuronal cell cycle reentry in a mouse model of Alzheimer disease. J. Clin. Investig..

[25] Dvir E., Elman A., Simmons D., Shapiro I., Duvdevani R., Dahan A., Hoffman A., Friedman J.E. (2007). DP-155, a lecithin derivative of indomethacin, is a novel nonsteroidal antiinflammatory drug for analgesia and Alzheimer’s disease therapy. CNS Drug Rev..

[26] Yamamoto T., Nozaki-Taguchi N. (1996). Analysis of the effects of cyclooxygenase (COX)-1 and COX-2 in spinal nociceptive transmission using indomethacin, a non-selective COX inhibitor, and NS-398, a COX-2 selective inhibitor. Brain Res..

[27] Smith W.L., DeWitt D.L., Garavito R.M. (2000). Cyclooxygenases: Structural, cellular, and molecular biology. Annu. Rev. Biochem..

[28] Wang P., Guan P.P., Wang T., Yu X., Guo J.J., Wang Z.Y. (2014). Aggravation of Alzheimer’s disease due to the COX-2-mediated reciprocal regulation of IL-1beta and Abeta between glial and neuron cells. Aging Cell.

[29] Wang P., Guan P.P., Guo J.W., Cao L.L., Xu G.B., Yu X., Wang Y., Wang Z.Y. (2016). Prostaglandin I2 upregulates the expression of anterior pharynx-defective-1alpha and anterior pharynx-defective-1beta in amyloid precursor protein/presenilin 1 transgenic mice. Aging Cell.

[30] Wang P., Guan P.P., Yu X., Zhang L.C., Su Y.N., Wang Z.Y. (2016). Prostaglandin I(2) Attenuates Prostaglandin E(2)-Stimulated Expression of Interferon gamma in a beta-Amyloid Protein- and NF-kappaB-Dependent Mechanism. Sci. Rep..

[31] Wang Y., Guan P.P., Yu X., Guo Y.S., Zhang Y.J., Wang Z.Y., Wang P. (2017). COX-2 metabolic products, the prostaglandin I2 and F2alpha, mediate the effects of TNF-alpha and Zn(2+) in stimulating the phosphorylation of Tau. Oncotarget.

[32] Misra U.K., Pizzo S.V. (2001). Induction of cyclooxygenase-2 synthesis by ligation of the macrophage alpha(2)-macroglobulin signalling receptor. Cell Signal.

[33] Hoffman M., Pizzo S.V., Weinberg J.B. (1988). Alpha 2 macroglobulin-proteinase complexes stimulate prostaglandin E2 synthesis by peritoneal macrophages. Agents Actions.

[34] Uhing R.J., Martenson C.H., Rubenstein D.S., Hollenbach P.W., Pizzo S.V. (1991). The exposure of murine macrophages to alpha 2-macroglobulin ‘fast’ forms results in the rapid secretion of eicosanoids. Biochim. Biophys. Acta.

[35] Sengupta S., Fine J., Wu-Wang C.Y., Gordon J., Murty V.L., Slomiany A., Slomiany B.L. (1990). The relationship of prostaglandins to cAMP, IgG, IgM and alpha-2-macroglobulin in gingival crevicular fluid in chronic adult periodontitis. Arch. Oral Biol..

[36] Narita M., Holtzman D.M., Schwartz A.L., Bu G. (1997). Alpha2-macroglobulin complexes with and mediates the endocytosis of beta-amyloid peptide via cell surface low-density lipoprotein receptor-related protein. J. Neurochem..

[37] Du Y., Bales K.R., Dodel R.C., Liu X., Glinn M.A., Horn J.W., Little S.P., Paul S.M. (1998). Alpha2-macroglobulin attenuates beta-amyloid peptide 1-40 fibril formation and associated neurotoxicity of cultured fetal rat cortical neurons. J. Neurochem..

[38] Williams S.E. (1992). A novel mechanism for controlling the activity of alpha 2-macroglobulin receptor/low density lipoprotein receptor-related protein. Multiple regulatory sites for 39-kDa receptor-associated protein. J. Biol. Chem..

[39] Padmasekar M., Nandigama R., Wartenberg M., Klaus-Dieter S., Sauer H. (2007). The acute phase protein α2-macroglobulin induces rat ventricular cardiomyocyte hypertrophy via ERK1,2 and PI3-kinase/Akt pathways. Cardiovasc. Res..

[40] Misra U.K., Pizzo S.V., Zoran C. (2012). Receptor-Recognized α2-Macroglobulin Binds to Cell Surface-Associated GRP78 and Activates mTORC1 and mTORC2 Signaling in Prostate Cancer Cells. PLoS ONE.

[41] Matthijs G., Marynen P. (1991). A deletion polymorphism in the human alpha-2-macroglobulin (A2M) gene. Nucleic Acids Res..

[42] Blacker D., Wilcox M.A., Laird N.M., Rodes L., Horvath S.M., Go R.C., Perry R., Watson B., Bassett S.S., McInnis M.G. (1998). Alpha-2 macroglobulin is genetically associated with Alzheimer disease. Nat. Genet..

[43] Dodel R.C., Bales K.R., Farlow M.R., Gasser T., Paul S.M., Du Y. (1999). Rapid detection of a pentanucleotide deletion polymorphism in the human alpha2-macroglobulin gene. Clin. Chem..

[44] Blennow K., Ricksten A., Prince J.A., Brookes A.J., Emahazion T., Wasslavik C., Bogdanovic N., Andreasen N., Botsman S., Marcusson J. (2000). No association between the α2-macroglobulin (A2M) deletion and Alzheimer’s disease, and no change in A2M mRNA, protein, or protein expression. J. Neural. Transm..

[45] Liao A., Nitsch R.M., Greenberg S.M., Finckh U., Blacker D., Albert M., Rebeck G.W., Gomez-Isla T., Clatworthy A., Binetti G. (1998). Genetic association of an alpha2-macroglobulin (Val1000lle) polymorphism and Alzheimer’s disease. Hum. Mol. Genet..

[46] Myllykangas L., Polvikoski T., Sulkava R., Verkkoniemi A., Crook R., Tienari P.J., Pusa A.K., Niinisto L., O’Brien P., Kontula K. (1999). Genetic association of alpha2-macroglobulin with Alzheimer’s disease in a Finnish elderly population. Ann. Neurol..

[47] Dow D.J., Lindsey N., Cairns N.J., Brayne C., Robinson D., Huppert F.A., Paykel E.S., Xuereb J., Wilcock G., Whittaker J.L. (1999). Alpha-2 macroglobulin polymorphism and Alzheimer disease risk in the UK. Nat. Genet..

[48] Rogaeva E.A., Premkumar S., Grubber J., Serneels L., Scott W.K., Kawarai T., Song Y., Hill D.L., Abou-Donia S.M., Martin E.R. (1999). An alpha-2-macroglobulin insertion-deletion polymorphism in Alzheimer disease. Nat. Genet..

[49] Alvarez V., Alvarez R., Lahoz C.H., Martinez C., Pena J., Guisasola L.M., Salas-Puig J., Moris G., Uria D., Menes B.B. (1999). Association between an alpha(2) macroglobulin DNA polymorphism and late-onset Alzheimer’s disease. Biochem. Biophys. Res. Commun..

[50] Bauer J., Strauss S., Schreiter-Gasser U., Ganter U., Schlegel P., Witt I., Yolk B., Berger M. (1991). Interleukin-6 and alpha-2-macroglobulin indicate an acute-phase state in Alzheimer’s disease cortices. FEBS Lett..

[51] Strauss S., Bauer J., Ganter U., Jonas U., Berger M., Volk B. (1992). Detection of interleukin-6 and alpha 2-macroglobulin immunoreactivity in cortex and hippocampus of Alzheimer’s disease patients. Lab. Investig..

[52] Van Gool D., De Strooper B., Van Leuven F., Triau E., Dom R. (1993). alpha 2-Macroglobulin expression in neuritic-type plaques in patients with Alzheimer’s disease. Neurobiol. Aging.

[53] Wood J.A., Wood P.L., Ryan R., Graff-Radford N.R., Pilapil C., Robitaille Y., Quirion R. (1993). Cytokine indices in Alzheimer’s temporal cortex: No changes in mature IL-1 beta or IL-1RA but increases in the associated acute phase proteins IL-6, alpha 2-macroglobulin and C-reactive protein. Brain Res..

[54] Boccalini G., Sassoli C., Bani D., Nistri S. (2018). Relaxin induces up-regulation of ADAM10 metalloprotease in RXFP1-expressing cells by PI3K/AKT signaling. Mol. Cell. Endocrinol..

[55] Leandro C.C., Bonacci G.R., Maria C.S., Chiabrando G.A. (2010). Activated α2 macroglobulin induces matrix metalloproteinase 9 expression by low-density lipoprotein receptor-related protein 1 through MAPK-ERK1/2 and NF-κB activation in macrophage-derived cell lines. J. Cell Biochem..

[56] (2002). Avramovich; Y, Non-steroidal Anti-inflammatory Drugs Stimulate Secretion of Non-amyloidogenic Precursor Protein. J. Biol. Chem..

[57] Wyatt A.R., Constantinescu P., Ecroyd H., Dobson C.M., Wilson M.R., Kumita J.R., Yerbury J.J. (2013). Protease-activated alpha-2-macroglobulin can inhibit amyloid formation via two distinct mechanisms. FEBS Lett..

[58] Deane R., Wu Z., Sagare A., Davis J., Du Yan S., Hamm K., Xu F., Parisi M., LaRue B., Hu H.W. (2004). LRP/amyloid beta-peptide interaction mediates differential brain efflux of Abeta isoforms. Neuron.

[59] Hollenbach E., Ackermann S., Hyman B.T., Rebeck G.W. (1998). Confirmation of an association between a polymorphism in exon 3 of the low-density lipoprotein receptor-related protein gene and Alzheimer’s disease. Neurology.

[60] Kang D.E., Saitoh T., Chen X., Xia Y., Masliah E., Hansen L.A., Thomas R.G., Thal L.J., Katzman R. (1997). Genetic association of the low-density lipoprotein receptor-related protein gene (LRP), an apolipoprotein E receptor, with late-onset Alzheimer’s disease. Neurology.

[61] Kang D.E., Pietrzik C.U., Baum L., Chevallier N., Merriam D.E., Kounnas M.Z., Wagner S.L., Troncoso J.C., Kawas C.H., Katzman R. (2000). Modulation of amyloid beta-protein clearance and Alzheimer’s disease susceptibility by the LDL receptor-related protein pathway. J. Clin. Investig..

[62] Shibata M., Yamada S., Kumar S.R., Calero M., Bading J., Frangione B., Holtzman D.M., Miller C.A., Strickland D.K., Ghiso J. (2000). Clearance of Alzheimer’s amyloid-ss(1-40) peptide from brain by LDL receptor-related protein-1 at the blood-brain barrier. J. Clin. Investig..

[63] Hsiao K., Chapman P., Nilsen S., Eckman C., Harigaya Y., Younkin S., Yang F., Cole G. (1996). Correlative memory deficits, Abeta elevation, and amyloid plaques in transgenic mice. Science.

[64] Storck S.E., Meister S., Nahrath J., Meiner J.N., Schubert N., Di S.A., Baches S., Vandenbroucke R.E., Bouter Y., Prikulis I. (2015). Endothelial LRP1 transports amyloid β1-42 across the blood-brain barrier. J. Clin. Investig..

[65] Kanekiyo T., Cirrito J.R., Liu C.-C., Shinohara M., Li J., Schuler D.R., Shinohara M., Holtzman D.M., Bu G. (2013). Neuronal clearance of amyloid-?2 by endocytic receptor LRP1. J. Neurosci..

[66] Pietrzik C.U., Busse T., Merriam D.E., Weggen S., Koo E.H. (2002). The cytoplasmic domain of the LDL receptor-related protein regulates multiple steps in APP processing. EMBO J..

[67] Ulery P.G., Beers J., Mikhailenko I., Tanzi R.E., Rebeck G.W., Hyman B.T., Strickland D.K. (2000). Modulation of beta-amyloid precursor protein processing by the low density lipoprotein receptor-related protein (LRP). Evidence that LRP contributes to the pathogenesis of Alzheimer’s disease. J. Biol. Chem..

[68] Zerbinatti C.V., Wozniak D.F., Cirrito J., Cam J.A., Osaka H., Bales K.R., Zhuo M., Paul S.M., Holtzman D.M., Bu G. (2004). Increased soluble amyloid-beta peptide and memory deficits in amyloid model mice overexpressing the low-density lipoprotein receptor-related protein. Proc. Natl. Acad. Sci. USA.

[69] Chakrabarty P., Herring A., Ceballos-Diaz C., Da S.P., Golde T.E. (2011). Hippocampal expression of murine TNF results in attenuation of amyloid deposition in vivo. Mol. Neurodegener..

[70] Meda L., Cassatella M., Szendrei G., Otvos L., Baron P., Villalba M., Ferrari D., Rossi F. (1995). Activation of microglial cells by β-amyloid protein andinterferon-γ. Nature.

[71] Savarin-Vuaillat C., Ransohoff R.M. (2007). Chemokines and chemokine receptors in neurological disease: Raise, retain, or reduce?. Neurotherapeutics.

[72] Mcgeer P.L., Mcgeer E.G. (2007). NSAIDs and Alzheimer disease: Epidemiological, animal model and clinical studies. Neurobiol. Aging.

[73] Xia M.Q., Qin S.X., Wu L.J., Mackay C.R., Hyman B.T. (1998). Immunohistochemical Study of the β-Chemokine Receptors CCR3 and CCR5 and Their Ligands in Normal and Alzheimer’s Disease Brains. Am. J. Pathol..

[74] Buchhave P., Minthon L., Zetterberg H., Wallin A.K., Blennow K., Hansson O. (2012). Cerebrospinal fluid levels of beta-amyloid 1-42, but not of tau, are fully changed already 5 to 10 years before the onset of Alzheimer dementia. Arch. Gen. Psychiatry.

[75] Prestia A., Caroli A., Van D., Ossenkoppele R., Berckel B.V., Barkhof F., Teunissen C.E., Wall A.E., Carter S.F., Scholl M. (2013). Prediction of dementia in MCI patients based on core diagnostic markers for Alzheimer disease. Neurology.

[76] Benito-León J., Contador I., Vega S., Villarejo-Galende A., Bermejo-Pareja F. (2019). Non-steroidal anti-inflammatory drugs use in older adults decreases risk of Alzheimer’s disease mortality. PLoS ONE.

[77] Hampel H., Caraci F., Cuello A.C., Caruso G., Lista S. (2020). A Path Toward Precision Medicine for Neuroinflammatory Mechanisms in Alzheimer’s Disease. Front. Immunol..

[78] Yu X., Guan P.-P., Guo J.-W., Wang Y., Cao L.-L., Xu G.-B., Konstantopoulos K., Wang Z.-Y., Wang P. (2015). By suppressing the expression of anterior pharynx-defective-1α and -1β and inhibiting the aggregation of β-amyloid protein, magnesium ions inhibit the cognitive decline of amyloid precursor protein/presenilin 1 transgenic mice. FASEB J..

[79] Wang P., Yu X., Guan P.-P., Guo J.-W., Wang Y., Zhang Y., Zhao H., Wang Z.-Y. (2017). Magnesium ion influx reduces neuroinflammation in Aβ2 precursor protein/Presenilin 1 transgenic mice by suppressing the expression of interleukin-1β2. Cell Mol. Immunol..

[80] Xu G.B., Yang L.Q., Guan P.P., Wang Z.Y., Wang P. (2019). Prostaglandin A1 Inhibits the Cognitive Decline of APP/PS1 Transgenic Mice via PPARγ/ABCA1-dependent Cholesterol Efflux Mechanisms. Neurotherapeutics.

[81] Halfmann R., Lindquist S. (2008). Screening for Amyloid Aggregation by Semi-Denaturing Detergent-Agarose Gel Electrophoresis. J. Vis. Exp..

[82] Lauer D., Reichenbach A., Birkenmeier G. (2001). α2-Macroglobulin-Mediated Degradation of Amyloid β1-42: A Mechanism to Enhance Amyloid β Catabolism. Exp. Neurol..

